# Humidity Sensing Behavior of Endohedral Li-Doped and Undoped SWCNT/SDBS Composite Films

**DOI:** 10.3390/s19010171

**Published:** 2019-01-05

**Authors:** Christian Müller, Ammar Al-Hamry, Olfa Kanoun, Mahfujur Rahaman, Dietrich R. T. Zahn, Elaine Yoshiko Matsubara, José Mauricio Rosolen

**Affiliations:** 1 Departamento de Física, Universidade Federal de Santa Catarina, Florianopolis 88040-900, Brazil; 2 Measurement and Sensor Technology, Chemnitz University of Technology, D-09107 Chemnitz, Germany; ammar.al-hamry@etit.tu-chemnitz.de (A.A.-H.); olfa.kanoun@etit.tu-chemnitz.de (O.K.); 3 Semiconductor Physics, Chemnitz University of Technology, D-09107 Chemnitz, Germany; mahfujur.rahaman@s2011.tu-chemnitz.de (M.R.); zahn@physik.tu-chemnitz.de (D.R.T.Z.); 4 Faculdade de Filosofia Ciencias e Letras de Ribeirão Preto-Departamento de Química-Universidade de São Paulo, Ribeirão Preto 14040-901, Brazil; elainematsubara@yahoo.com (E.Y.M.); rosolen@ffclrp.usp.br (J.M.R.)

**Keywords:** carbon nanotubes, thin films, humidity sensor, temperature sensor, impedance spectroscopy, Raman spectroscopy, electron microscopy

## Abstract

We have investigated single-walled carbon nanotube (SWCNT) networks wrapped with the cationic surfactant sodium dodecyl-benzenesulfonate (SBDS) as promising candidates for water detection. This is the first time that the humidity behavior of endohedral Li-doped (Li@) and undoped SWCNTs/SDBS has been shown. We identified a strong and almost monotonic decrease in resistance as humidity increased from 11 to 97%. Sensitivities varied between −3 and 65% in the entire humidity range. Electrical characterization, Raman spectroscopy, and high-resolution transmission electron microscopy (HRTEM) analysis revealed that a combination of the electron donor behavior of the water molecules with Poole-Frenkel conduction accounted for the resistive humidity response in the Li@SWCNT/SDBS and undoped SWCNT/SDBS networks. We found that Li@SWCNTs boosted the semiconducting character in mixtures of metallic/semiconducting SWCNT beams. Moreover, electrical characterization of the sensor suggested that endohedral Li doping produced SWCNT beams with high concentration of semiconducting tubes. We also investigated how frequency influenced film humidity sensing behavior and how this behavior of SWCNT/SDBS films depended on temperature from 20 to 80 ${}^{{}^{\circ}}$C. The present results will certainly aid design and optimization of SWCNT films with different dopants for humidity or gas sensing in general.

## 1. Introduction

Humidity sensors are highly important in many industrial fields, such as automobile industry, environmental monitoring, food production, health monitoring and production processes [1,2,3]. To date, different materials, including carbon nanotubes (CNTs) [4,5], graphene [6], graphene oxide [7,8], carbon nitride [9], metal oxides [10], polymers and nanocomposites [11,12], have been explored for humidity sensing. Among these materials, CNTs have gained great interest because of their excellent mechanical robustness, electrical conductivity, and chemical stability [13]. High amount of surface atoms and large aspect ratio make CNTs ideal candidates for humidity and gas sensors [14]. Typically CNT based humidity sensors are fabricated by chemical vapor deposition [15], Langmuir–Blodgett technique [16], self-assembly [17], drop casting [18], electrophoretic deposition [19], and printing techniques [20]. Among these preparation techniques, techniques that use liquid CNT dispersions require surfactants to ensure a good dispersion quality [21]. While most studies have focused on multi-walled CNTs, the way humidity impacts SWCNT electrical conductivity has rarely been investiated [22,23,24,25]. Zahab et al. [22] reported that water molecules can act as SWCNT dopants to modify their electrical conductivity. In fact, concerning the area of sensors, SWCNTs are particularly attractive because they provide ballistic electron transport and have excellent ability to exchange charge with several chemical species. The way surfactants impact the electrical properties of SWCNTs has recently been studied [26]. In the presence of cationic surfactants, conductance increases upon exposure to water; as for anionic surfactants, the opposite effect arises. The change of conductance was mainly attributed to electrostatic interactions of water molecules with charged head groups and the counterion of the surfactant, which induce an electrostatic potential on the SWCNTs. In another work [27] it was found, that SWCNT film electrical conductance significantly changes in the presence of surfactants because they block inter-nanotube connections in the CNT network and enhance contact resistance [28,29]. Moreover, the frequency response of CNT based humidity sensors can give more insights about the sensor performance and conduction behavior [30]. For instance, Zhao et al. [31] found that the applied frequency was directly related to the multi-walled CNT (MWCNT) humidity sensor sensitivity and stability. However, only a few works have focused on SWCNT response to frequency [31,32] and, to our knowledge, there are no reports on the use Li@SWCNTs for humidity sensing. In case of Li@SWCNTs, improved electrical transport properties and taylored ratios between semiconducting and metallic nanotubes could be achieved. In particular, doping with Li would help to shift the electrical properties of SWCNTs to more semiconducting behavior. Investigation into the humidity sensing behavior of SWCNT films could provide deeper knowledge about their physical properties and enable the preparation of sensors with desired characteristics, like high sensitivity, large measurement range, stability, fast response, good reproducibility, small hysteresis, and linearity. Knowledge about SWCNT film properties could help to predict their potential in more complex sensor materials, particularly in materials containing functionalized SWCNTs or SWCNTs in composites. Therefore, this work aimed to evaluate the humidity sensing behavior of SWCNT/SBDS and Li@SWCNT/SDBS thin films and to assess the role played by the surfactant SDBS and Li@SWCNT, temperature response, and frequency response in film properties.

## 2. Materials and Methods

### 2.1. CNT Fabrication

Li@SWCNT and SWCNT samples were prepared by the arc discharge method; NiO/CoO (molar ratio 1:1) and Li${}_{2}$CO${}_{3}$/NiO/CoO (molar ratio 2:1:1) were used as catalysts, respectively. The arc-reactor operated at a current of 120 A and pressure of 400 mTorr He. Li atoms were encapsulated inside the SWCNTs, as previously reported by Matsubara et al. [33]. More specifically, Li was inserted into the closed hollow tubes during SWCNT growth. In fact, Li@SWCNT preparation involved a step during which the tubes were rinsed to remove Li that was located between or on the surface of the tubes. The Li@SWCNTs employed in the present study were stable in water: their zeta potential was −37 mV, whereas the zeta potential of SWCNTs prepared only with NiO/CoO is −25 mV [33].

### 2.2. Film Preparation

**Figure 1:**
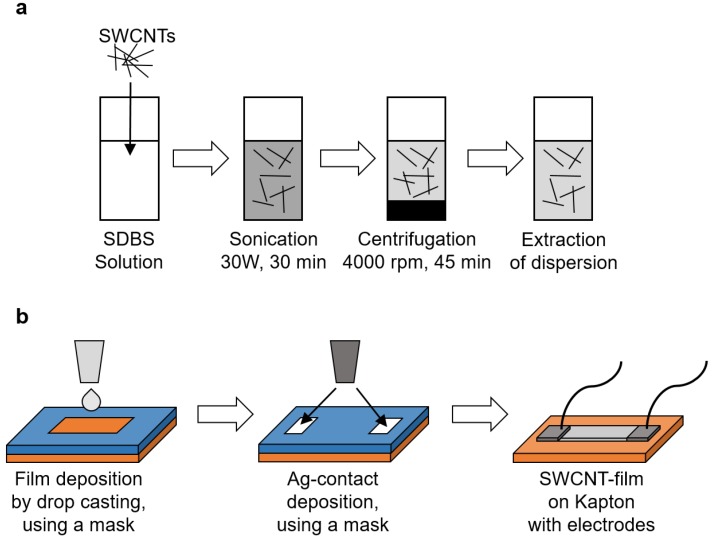
Film fabrication process: (**a**) Dispersion preparation and (**b**) Film preparation.

To stabilize SWCNTs in water the anionic surfactant SBDS) which consists of a hydrophilic sulfonate head group and a hydrophobic alkylbenzene group, was used. Initially, SBDS aqueous solution (50 g) was prepared by mixing 0.5 g of SDBS with 49.5 g of DI water (1% SDBS aqueous solution). This mixture was magnetically stirred at 1000 rpm for 15 min. Then, 0.05 wt.% of CNTs (either SWCNTs or Li@SWCNTs) were dispersed in the SDBS aqueous solution. Next, the resulting aqueous SWCNT/SDBS and Li@SWCNTs/SDBS dispersions were sonicated with a spherical tip at 25 ${}^{{}^{\circ}}$C for 30 min to ensure that the SWCNTs were well distributed in the aqueous phase. Sonication power amplitude of 30 W with square pulses (0.5 s active and 0.5 s passive) was applied with a Bandelin Solopuls HD 3200 sonicator. After sonication, the dispersions were centrifuged at 4000 rpm for 45 min in a Sigma 2-16PK centrifugation chamber. The dispersed SWNTs (clear part of the dispersion) were extracted after centrifugation and considered for thin film deposition. Before deposition, the Kapton foil substrate was cut into 5 cm × 2.5 cm slices, carefully cleaned with ethanol and DI water, and dried with nitrogen. Then, the Kapton substrate was covered with a mask foil, a square pattern of 1.2 cm × 0.6 cm was opened on the mask foil, and 25 μL of the sonicated SWNT dispersions per layer was deposited by drop casting on the open pattern with an electronic pipette. After each drop casting step, films were dried at 45 ${}^{{}^{\circ}}$C for 20 min in air. This procedure was repeated until five layers had been deposited by drop casting. The total layer thickness amounted to ≈5 μm. After drying, the mask foil was carefully removed from the substrate, and Ag-electrodes (thickness ≈ 1 μm) were made at both ends of film. Finally, cables were connected to the electrodes with silicone epoxy and allowed to dry overnight. Figure 1 summarizes the SWCNT fabrication scheme.

### 2.3. Material Characterization

**Figure 2:**
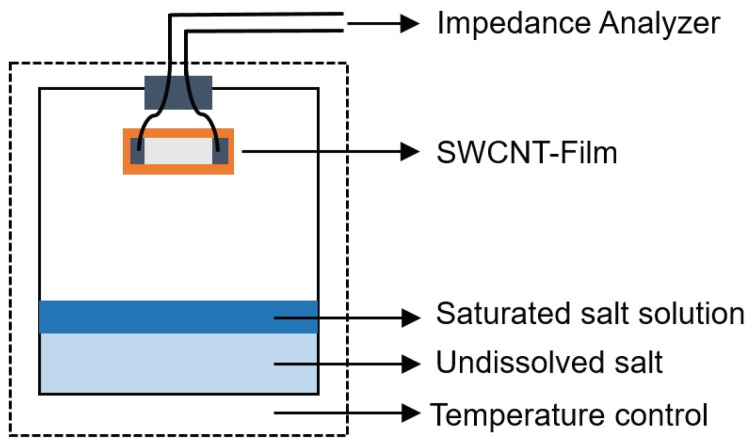
Experimental setup for humidity measurements.

To try to understand how the SWCNTs bind to SDBS water after evaporation, some drops of SWCNT/SDBS dispersion were deposited on Si substrate and analyzed by electron scanning microscopy (SEM) using a Nova nano 450 SEM (FEI, Hillsboro, OR, USA). In addition, high resolution transmission electron microscopy (HRTEM) analysis was performed with a JEM 3010 TEM operating at 300 kV (JEOL, Tokyo, Japan). For analysis a small drop of the resulting dispersion were placed onto lacey-carbon/copper grids (300 mesh). The structure and electronic properties of the dispersed SWCNTs were also investigated by Micro-Raman spectroscopy. A LabRam HR800 micro-Raman system (JY Horiba, Kyoto, Japan) equipped with liquid nitrogen-cooled CCD was used for the Raman measurement. The Li@SWCNTs and SWCNTs dispersed in water were deposited on Si substrate and were excited with a 514.7 nm (2.41 eV) diode pumped solid-state laser (Cobolt AB, Solna, Sweden) by using 100×, 0.9 N.A. objective, and the scattered signals were dispersed by 600 L/mm grating onto the detector. The laser power measured at the sample surface was 167 mW. For temperature investigations, changes in the electrical resistance of the samples as a function of the temperature were measured by applying a cyclic temperature profile from 20 to 80 ${}^{{}^{\circ}}$C and back to 20 ${}^{{}^{\circ}}$C, with constant heating and cooling rate of 30 ${}^{{}^{\circ}}$C/h; a Vötsch VT4002 temperature test chamber was used. At each temperature, resistance was measured from −0.5 to 0.5 V with the aid of a Keithley 2602 dual-channel source meter (Keithley, Cleveland, OH, United States). Humidity experiments were carried out in a custom-designed setup by using different saturated salt solutions and a constant temperature of 20 ${}^{{}^{\circ}}$C (see Figure 2). In our experiments, lithium chloride (LiCl—11%), magnesium chloride (MgCl${}_{2}$—33%), sodium chloride (NaCl—75.6%), potassium chloride (KCl—84%), and potassium sulfate (K${}_{2}$SO${}_{4}$—97.3%) were used as drying agents. Then, under defined humidity DC resistance, measurements were performed with an Agilent 34,401 A digital multimeter (Agilent Technologies, Santa Clara, CA, USA), which was controlled by a LabVIEW program. Next, AC impedance was measured with an Agilent 4294 A impedance analyzer working between 40 Hz and 110 MHz. The obtained AC impedance data were represented by using Bode [34] and Nyquist plots [35].

## 3. Results and Discussion

### 3.1. Raman Spectroscopy

**Figure 3:**
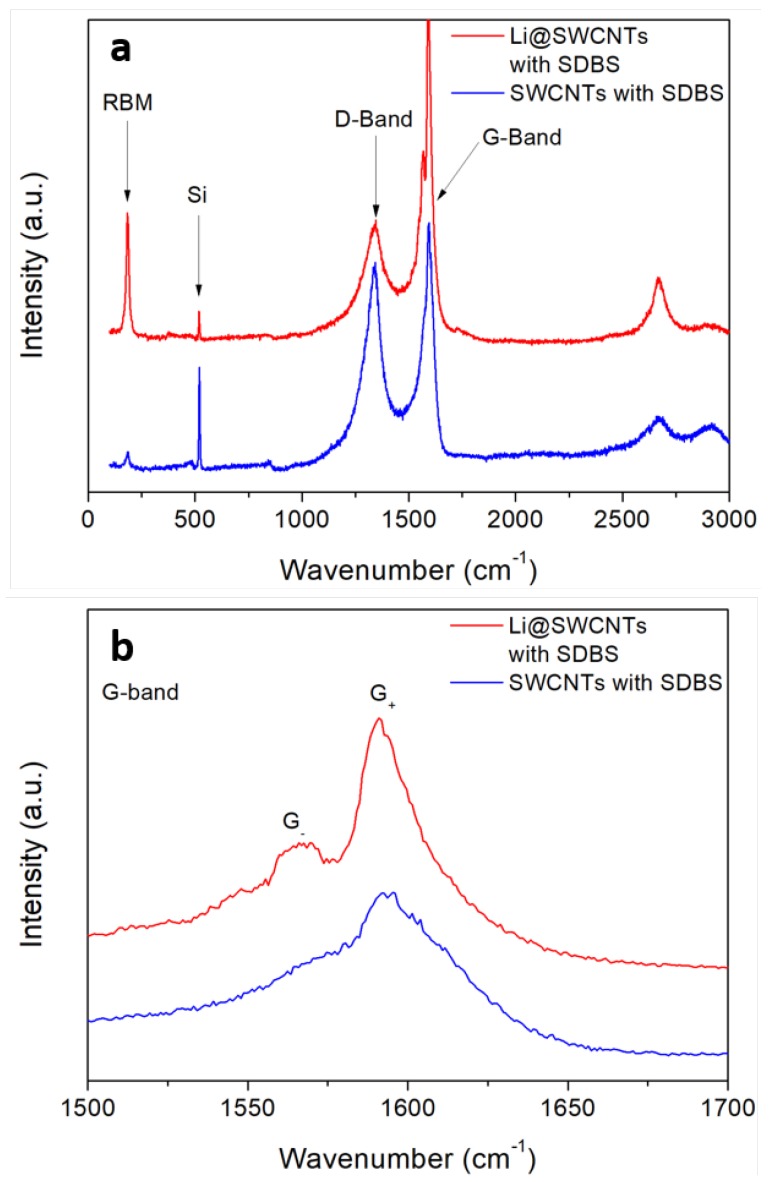
Raman spectra of undoped SWCNT and Li@SWCNTs dispersed with SDBS and deposited on silicon. (**a**) Overview spectra (**b**) Detailed view of the G-band.

We analyzed the structural properties of SWCNTs and Li@SWCNTs dispersed in SBDS by Raman spectroscopy (Figure 3). The Raman spectra confirmed the effective presence of SWCNTs, as expected. However, the Li@SWCNT predominant radial breathing mode (RBM) was more intense as compared to undoped SWCNTs. Detailed Raman analysis of these same samples excited at 632.8 nm revealed a similar behavior [33]. When excited at 632.8 or 514 nm, Li@SWCNTs had narrower tube diameter distribution than undoped SWCNTs. Taking into account the relationship between the radial vibration wavenumber and the inverse of the CNT tube diameter as $\omega_{RBM}=C_{1}/d_{CNT}+C_{2}$ [36] and setting $C_{1}$ as 248 cm${}^{-1}$ and $C_{2}$ as 0, the predominant Li@SWCNT diameter was about 1.35 nm. However, the D and G bands were the most important aspects of Li@SWCNTs and undoped SWCNTs employed during preparation of SBDS composite film sensors. The band around 1337 cm${}^{-1}$ is known as the D band (defect-induced) and is based on structural defects in the carbon lattice. As for the G band, which is assigned to carbon structures with $sp^{2}$ hybridization, splitting into G${}_{+}$ and G${}_{-}$ peaks centered around 1592 and 1565 cm${}^{-1}$, respectively, occurred. Nevertheless, this splitting was more pronounced for Li@SWCNTs, whereas the spectrum of undoped SWCNTs displayed only a small shoulder. Such peak splitting is typical of SWCNTs, and it is well known that the G band profile is sensitive to the concentration of semiconducting or metallic tubes in the SWCNT ropes. In general, SWCNTs with high metallic tube concentration present a G${}_{-}$ band associated with vibrations along the circumferential direction, which is expected to be broader and more intense than the G${}_{+}$ band. This can be observed in the SWCNT Raman spectrum shown in [33] when SWCNTs were doped with endohedral and interstitial Li. Thus, the Li@SWCNT G band line shape could indicate that Li@SWCNT/SBDS films contained higher concentration of semiconducting SWCNT tubes than SWCNT/SBDS films. Moreover, Li@SWCNTs have better crystallinity or smaller concentration of structural defects as compared to undoped SWCNTs due to lower D and G${}_{+}$ peak with $I_{D}$/$I_{G_{+}}$ = 0.34 and 0.84 for Li@SWCNTs and undoped SWCNTs, respectively.

### 3.2. Electron Microscopy

**Figure 4:**
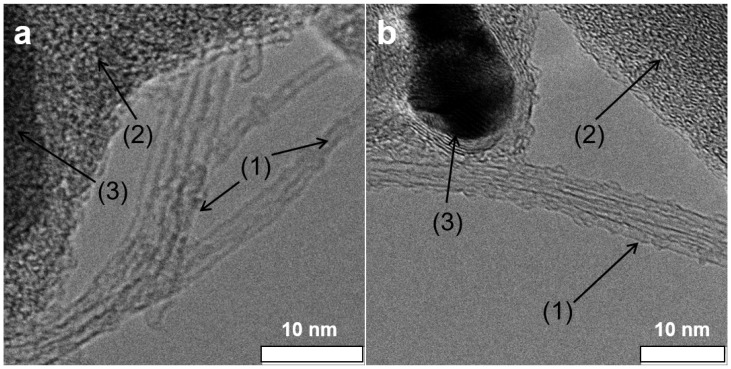
High resolution transmission electron microscopy images of (**a**) undoped SWCNTs and (**b**) Li@SWCNTs washed in deionized water (1). Besides the tubes, samples contained amorphous carbon (2) and core-shell structures of catalyst particles (3).

TEM analysis of the as-grown material attested to the presence of SWCNTs besides catalyst particles covered with a carbon shell (see Figure 4). Li@SWCNT tube diameter was estimated to be 1.3 nm, in agreement with the Raman spectroscopy results. Figure 4 illustrates HRTEM analysis of the Li@SWCNTs and SWCNTs that we used to construct the sensor films. The HRTEM pictures confirmed the Raman spectral findings. The Li@SWCNT beams contained tubes with less disordered walls than undoped tubes. The average Li@SWCNT tube diameter determined by HRTEM was 1.35 nm, which agreed with Raman spectroscopy data. Undoped SWCNTs were a little smaller (1.31 nm). The doped and undoped SWCNT beam lengths ranged from 1 to 10 μm. HRTEM analyses also proved that both Li@SWCNTs and undoped SWCNTs contained amorphous carbon and graphitic core-shell structures, which constitute common impurities in non-purified CNTs [37].

**Figure 5:**
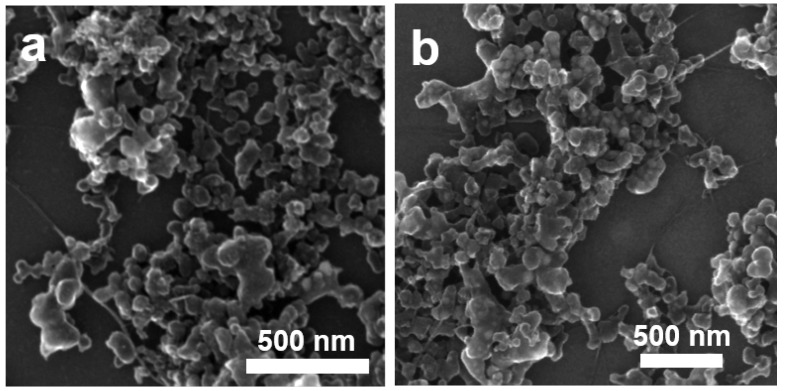
Scanning electron microscopy images of (**a**) undoped SWCNT/SDBS films and (**b**) of Li@SWCNT/SDBS films.

To understand what kind of morphology we could find in the sensor we prepared as described in Figure 2, we analyzed the undoped SWCNT/SBDS and Li@SWCNT/SDBS composites deposited on silicon by SEM. Both undoped SWCNT/SBDS and Li@SWCNT/SBDS composites show networks of irregular SBDS nanoparticles with size of around 100 nm. The agglomerated SBDS particles were interconnected by the micrometric SWCNT beams, which were well dispersed in the sample (Figure 5). Therefore, undoped SWCNT/SBDS and Li@SWCNT/SBDS films should form membranes on the kapton substrate containing networks of well-dispersed SWCNTs.

### 3.3. Electrical Measurements

**Figure 6:**
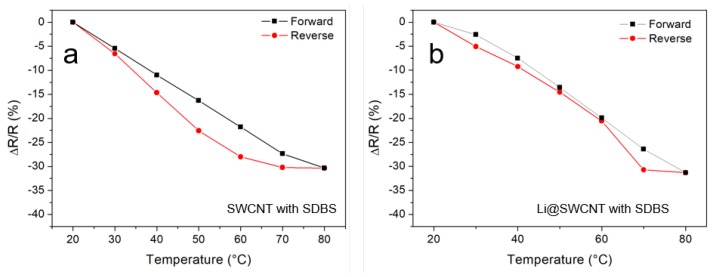
Temperature response of (**a**) of undoped SWCNT/SDBS films and (**b**) of Li@SWCNT/SDBS films. Measurement were performed at constant humidity of ≈10%.

As shown in Figure 6, resistance dependence on temperature for undoped SWCNT/SDBS and Li@SWCNT/SDBS films can be reversed. Both samples displayed a similar general behavior with almost the same relative changes in resistance. This general behavior was observed over several cycles (not shown). However, a small hysteresis was visible with maximum resistance difference between the forward (20–80 ${}^{{}^{\circ}}$C) and reverse (8–20 ${}^{{}^{\circ}}$C) measurements of 6.2 and 4.3% for undoped SWCNT/SDBS and Li@SWCNT/SDBS films, respectively. Here SWCNT/SDBS films also exhibited a negative temperature coefficient (NTC) behavior with average temperature coefficient of −34 kΩ/K and −1.1 kΩ/K, for undoped SWCNT/SDBS and Li@SWCNT/SDBS films, respectively. The observed resistance dependence on temperature is typical of semiconducting SWCNTs [38].

**Figure 7:**
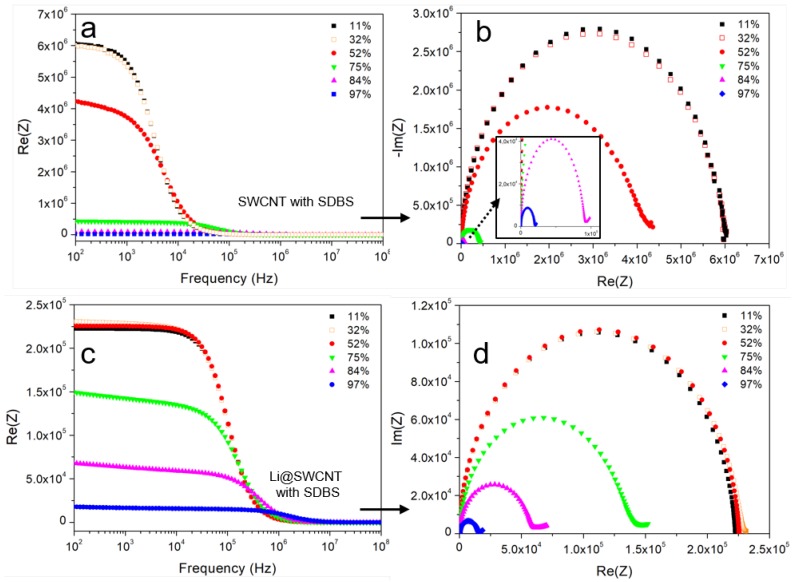
Impedance spectra in the humidity range of 11–97% of: (**a**) Bode plot of undoped SWCNT/SDBS films. (**b**) Nyquist plot of undoped SWCNT/SDBS films. (**c**) Bode plot of Li@SWCNT/SDBS films. (**d**) Nyquist plot of Li@SWCNT/SDBS films.

**Figure 8:**
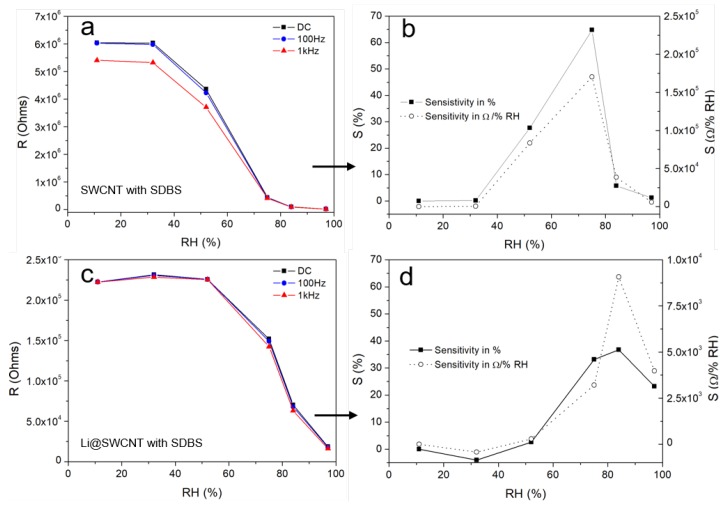
Resistance as a function of humidity taken at 0 Hz, 100 Hz, and 1 kHz and corresponding sensitivity at 0 Hz: of (**a**,**b**) undoped SWCNT/SDBS films and (**c**,**d**) Li@SWCNT/SDBS films.

SWCNT/SDBS films can form conductive networks when the SWCNT concentration is close to the percolation threshold or when sufficiently thick layers are fabricated. Here, we obtained conductive films after five layers were deposited from SWCNT/SDBS dispersions. Electrical impedance spectra of the SWCNT/SDBS films were recorded in an attempt to understand film electrical transport. Figure 7 shows that the overall impedance strongly depended on frequency and humidity. In general, undoped SWCNT/SDBS and Li@SWCNT/SDBS films presented resistive (at low frequencies) and capacitive (at high frequencies) regimes in their Bode plots. In undoped SWCNT/SDBS films exposed to humidities < 75%, transition between the resistive and capacitive regimes occurs at lower frequencies as compared to Li@SWCNT/SDBS films. This behavior indicated the presence of higher conductive paths in Li@SWCNT/SDBS than in SWCNT/SDBS films. The latter fact was also supported by one order of magnitude lower initial resistance values (at low frequencies). Analysis of the SWCNT network helped to understand the observed resistive and capacitive regions. Basically, each network nanotube had resistance $R_{CNT}$. Dispersion of undoped or Li-doped SWCNTs in SDBS provided a coating around the SWCNTs, which induced contact resistance $R_{C}$ and capacitance $C_{Gap}$ between neighboring tubes. Therefore, the initial resistance values of the two sample types most probably originated from different SWCNT $R_{CNT}$ and $R_{C}$ in the network due to inter-tube distance variation. When we observed the humidity dependence on both SWCNT film types, we verified that the overall resistance dropped sharply over the whole measurement range (Figure 8). According to the applied humidity steps, we calculated film sensitivities (see Figure 8) by using:

(1) $$S=\frac{R_{i}-R_{j}}{\mathrm{RH}(10\%)}\cdot 100\%$$

where $R_{i}$ and $R_{j}$ are the resistances of neighbouring points in the resistance-humidity plot and RH(10%) corresponds to the resistance measured at 10% humidity. We were able to identify three regions with different sensitivity within the entire humidity range. At low humidities (<32% RH), overall resistance remained almost constant, indicating small changes in $R_{CNT}$ and $R_{C}$ in the SWCNT/SDBS network. Overall resistance (high sensitivity) dropped markedly from 32 to 75% RH and from 32 to 84% RH for undoped SWCNT/SDBS and Li@SWCNT/SDBS films, respectively. From the sensitivities shown in Figure 8 resistance changes of up to ≈3%/%RH and ≈4%/%RH were calculated for undoped SWCNT/SDBS and Li@SWCNT/SDBS films, respectively. These values are equal or slightly higher than reported for resistive humidity sensors based on other materials, for example, graphene (0.31%/%RH [6]), MWCNT films (up to 1.3%/%RH [39]), MWCNT/PEDOT:PSS composites (≈1.1%/%RH [12]), SWCNTs (≈3%/%RH [40]), SnO${}_{2}$ (≈2–3%/%RH [41]). We assumed that strong interaction between water molecules and SWCNTs/SDBS induced the resistance change in our samples. In the presence of sufficient water, SDBS molecules adsorbed on the nanotube surface could be rearranged in such a way that the hydrophilic ends were oriented away from the nanotubes [26,42]. This rearrangement would enable water molecules to interact with the SWCNTs and allow electrons to hop from the water molecules to the SWCNTs. Because there was a mixture of semiconducting and metallic SWCNTs in both kinds of composite films, excess semiconducting SWCNTs resulted in electrical properties being governed by the semiconducting nanotubes. In fact, this agreed with the CNT temperature behavior (see Figure 6). The adsorbed water molecules acted as electron donors in a p-type semiconductor. Low humidity compensated the holes in the semiconducting tubes, so change in resistance was small. After hole compensation, SWCNTs behaved as n-type semiconductors. Compared to undoped SWCNTs, Li@SWCNTs contained a larger amount of semiconducting CNTs, so a higher quantity of water was necessary to compensate for the p-type character and to turn them into n-type semiconductors. Hence, resistance started to decrease at higher humidity. Similar resistance behavior has been observed in SWCNT mats [22] and in SWCNT networks containing a low amount of inter-tube junctions [43]. In addition, water molecules could adsorb onto site defect in SWCNTs and reduce resistance by Poole-Frenkel conduction [44,45]. Raman spectroscopy (Figure 3) and TEM analysis (Figure 4) proved the presence of site defects. Undoped SWCNTs had more site defects than Li@SWCNTs and could be another reason for the higher sensitivity in the humidity range from 32 to 52%. On the other hand, SWCNT/SDBS network swelling could occur. However, this would increase inter-tube distances with higher $R_{CNT}$ and would contradict our results, so it must have played a minor role. Consequently, the major change in the overall SWCNT/SDBS composite resistance arise from $R_{CNT}$ variations. Finally, at high humidities (>84% RH), the SWCNT/SDBS films were covered with a water film. In this situation, most of the SWCNT surface would be covered with water molecules, and further water addition would elicit a small or no change in resistance. Our results differ from the results recently reported by Evans et al. [26], who used SWCNTs in combination with anionic and cationic surfactants. It is also important to mention, that the SWCNT fabrication process and the film fabrication parameters (e.g., film thickness, type of SWCNTs, substrate type of surfactant) in ref. [26] differed from the parameters of the present work. Evans et al. [26] reported a drop in conductance for semiconducting SWCNTs in the presence of water when they used anionic surfactants (e.g., sodium deoxycholate (DOC), sodium cholate (SC)) due to electrostatic interactions inducing an increase in the p-type nature of semiconducting SWCNTs. In the present work electrostatic interactions do not dominate the conduction behavior. However, it can not be ruled out that the conductance drop in our films appearing at high humidities (>75% RH) is a result of increasing electrostatic interactions.

## 4. Conclusions

We found a strong humidity response for both undoped SWCNT and Li@SWCNT thin films combined with the anionic surfactant SDBS. The resistance of both film types drastically dropped at humidities >32%, which was attributed to water molecule electron donor behavior and to Poole-Frenkel conduction water adsorption onto site defects. Electrical impedance spectroscopy of the SWCNT/SDBS films provided insight into sensor performance and conduction behavior as a function of frequency and humidity. Depending on SWCNT type (undoped or Li-doped), we were able to assess different humidity ranges of up to 97% RH. We attributed differences in resistance-humidity characteristics of undoped SWCNT/SDBS and Li@SWCNT/SDBS films to different concentrations of metallic/semiconducting CNTs, varying crystallinity, and different number of site defects. Additionally, the use of Li@SWCNTs allowed us to obtain CNT networks with better electrical conductivity and increase the concentration of semiconducting tubes in mixtures of metallic and semiconducting tubes, issuing the problem of separating CNTs of different types. In agreement with previous works, the presence of surfactants can significantly change SWCNT films resistance upon exposure to water and give reversible humidity sensing behavior. However, the role played by anionic and cationic surfactants in SWCNT networks remains controversial. In fact, many film parameters, such as SWCNT material properties, orientation in the network, concentration, and film thickness, to name only a few, strongly impact the humidity sensing behavior. Therefore, deeper analysis of the aforementioned film parameter and the underlying mechanism are necessary to fabricate humidity sensors based on SWCNTs and SBDS with the desired properties. We expect that SWCNT and Li@SWCNT thin films will contribute to the development of novel humidity sensors because of their high sensitivity and low fabrication costs.
